# EMS: Efficient Monitoring System to Detect Non-Cooperative Nodes in IoT-Based Vehicular Delay Tolerant Networks (VDTNs)

**DOI:** 10.3390/s23010099

**Published:** 2022-12-22

**Authors:** Ghani Ur Rehman, Muhammad Zubair, Iqbal Qasim, Afzal Badshah, Zafar Mahmood, Muhammad Aslam, Syeda Fizah Jilani

**Affiliations:** 1 Department of Computer Science and Bioinformatics, Khushal Khan Khattak University, Karak 27000, Pakistan; 2 Department of Computer Science, University of Science and Technology Bannu, Bannu 28100, Pakistan; 3 Department of Computer Science & Software Engineering, International Islamic University, Islamabad 44000, Pakistan; 4 Department of Computer Science, University of Gujrat, Gujrat 50700, Pakistan; 5 School of Computing Engineering and Physical Sciences, University of West of Scotland, Glasgow G72 0LH, UK; 6 Scotland Academy, Wuxi Taihu University, Wuxi 214063, China; 7 Department of Physics, Physical Sciences Building, Aberystwyth University, Aberystwyth SY23 3BZ, UK

**Keywords:** Internet of Things, VDTNs, selfish nodes, monitoring system, cooperation

## Abstract

Since several Internet of Things (IoT) applications have been widely deployed on unstable wireless networks, such as the Delay Tolerant Network (DTN), data communication efficiency in DTN remains a challenge for IoT applications. Vehicular Delay Tolerant Network (VDTN) has become one of DTN’s potential applications, in which the network experiences connectivity interruption due to the lack of an end-to-end relay route. VDTNs focus on node cooperation to achieve this goal. As a result, it is essential to ensure that almost all network nodes adopt the protocol to preserve network performance. This is a challenging task since nodes may diverge from the basic protocol to optimize their effectiveness. This article presents an Efficient Monitoring System (EMS) to detect and respond to just selfish nodes to minimize their entire network and data communication efficacy. The scheme is based on a network-wide cooperative sharing of node reputation. It is also necessary to increase overall network efficiency by tracking selfish nodes. The NS-2 simulator is used to run this experimental setup. Simulation results indicate that the proposed scheme performs better in terms of probability of package delivery, package delivery delay, energy consumption, and amount of packet drops. For 80% selfish nodes in the network, the packet delivery of EMS is 37% and 31% better than SOS and IPS. Similarly, the average delivery delay of EMS is 22% and 18% lower than SOS and IPS when 80% selfish nodes are incorporated in the network.

## 1. Introduction

The Internet of Things (IoT) [1] is a new paradigm that involves connecting hand-held gadgets and everyday devices with sensing, computing, and communication functionality to create a network. Object recognition and monitoring, sensing information visualization, security control [2,3], object networking, and other fields of IoT research are all included [4]. With the introduction of IoT, existing networks have become more important for data transfer in IoT applications. On the other hand, several IoT applications are affected by issues such as insecure wireless, a poorly constructed trust model, and poor mobile networks. Lack of connectivity, diverse mobility, and prolonged connection disruptions are common characteristics of IoT systems in urban environments. Such unorganized networks are a subset of delay/Disruption Tolerant Networks (DTNs) [5,6,7]. End-to-end communication between sender and receiver nodes is rare in DTN. If the source node wants to send messages to the destination node, messages should always be transferred to intermediate participating nodes using the “store-carry-forward” method due to the interruption of communication. Furthermore, the time available for intermediate participating nodes to send messages is minimal. That is why the conventional routing protocols are ineffective in the “store-carry-forward” framework. As a result, developing effective DTN routing protocols in several IoT applications presents a serious challenge. One of the most popular forms of DTN is Vehicular Delay Tolerant Networks (VDTNs). VDTNs [8] are a new type of vehicular network whose architecture enables connectivity in situations in which an end-to-end route between the sender and receiver is not possible. VDTNs, like so many other ad hoc networks, depend on the cooperation and coordination among mobile network nodes that are used to receive, keep, carry, and forward packets [9,10].

There are three types of nodes in VDTNs, namely, terminal, relay, and mobile nodes. Terminal nodes are generally located only at the network’s boundary, which is in charge of massive data processing and connectivity with all other networks, e.g., the internet. The nodes that are located at road intersections are known as relay nodes. These nodes expand the number of network interactions and provide a larger amount of data packets that could be obtained by mobile vehicles in the range. The mobile nodes take routes and can communicate with the other types of nodes. Unlike other vehicular networks, VDTNs use two stages to address each communication opportunity: the control plane and the data plane (providing out-of-band signaling). In the initial phase of a communication opening by using the control plane, the nodes interchange preliminary information, such as buffer status, mobility speed, and destination node to establish and maintain services for proper data package delivery at the data plane. Messages are combined in the data plane and routed to either a single or several receivers. This out-of-band signaling technique permits various network technologies to be used in each plane and significantly increases network efficiency because nodes can decline a communication opportunity depending on the signaling information, and in general, that would keep resources or avoid data from being tampered with.

Despite all of the progress made, VDTNs are still coping with the existence of misbehaving nodes that do not adopt the specifications and harm the overall network results. Typically, this type of node takes advantage of and utilizes the services of several other nodes to serve their interests. The selfish node, for instance, is the one that drops packages without sending them at least once [11,12]. This type of node often wastes a lot of network resources and, therefore, can impact the efficiency of well-behaved nodes. This situation necessitates detecting certain nodes as well as taking measures toward them. Nevertheless, due to the mobility of vehicles, which enhances the uncertainty of their classification and identification, this is indeed a complex job. One useful strategy is to provide nodes with intelligent frameworks that can identify and prevent nodes that behave suspiciously [13,14,15].

An Efficient Monitoring System (EMS) is introduced in this article to facilitate network nodes in recognizing selfish nodes. EMS provides a reputation value to every network node to accomplish this goal. As a result, whenever nodes actively participate in a communication activity, the EMS adjusts their reputation value depending on four subsystems (nodes categorization, neighborhood assessment, punishment, and recommendation). The categorization component’s objective is to classify nodes into various types based on their reputation value. The categorization mechanism computes each node’s cooperation value by relying on their classification. The cooperation value is sent to the determination component, which uses it to penalize or incentivize the nodes based on how cooperative and collaborative they are. The neighbor’s assessment system handles how neighbors measure a node’s network reputation. This is done by seeking information regarding their views on this. The punishment component punishes the selfish nodes for showing selfish behavior regularly and blacklist such nodes into the network. The recommendation component adjusts the nodes’ reputation value and relies on the information exchanged by any of the other components after a communication opportunity. The EMS can categorize, track, and take action against such types of nodes. Whenever a selfish or non-cooperative node is identified, the EMS broadcasts an alert to all the neighbors of the node, enabling the message to circulate across the network. This alert can notify cooperative nodes that a newly selfish node has entered the network. The following are the essential points of this article:

- A summary of the most common credit-based, reputation-based, tit-for-tat-based, and hybrid-based cooperative communication approaches in vehicular networks.
- A case study demonstrating the negative effect of selfish and misbehavior nodes on VDTN efficiency using the package delivery probability, packet average delay, energy consumption, and amount of dropped packages as the evaluation criteria.
- The framework of an EMS is made up of three distinct components to detect and elude selfish nodes to minimize their effect on cooperative node outcomes.
- Analysis of the presented EMS solution’s effect on VDTN efficiency in terms of package delivery probability, package average delay, energy consumption, and the amount of dropped packets.

The remaining article is organized into the following different sections. The overview of existing incentive techniques is presented in Section 2. Section 3 provides a discussion to illustrate the effect of selfish nodes inside a VDTN network. The efficient monitoring system and its implementation in VDTNs are described in Section 4. In Section 5, the experimental results are presented. Lastly, the article is concluded, and future works are discussed in Section 6.

## 2. Related Works

The automobile and research communities have been directly contributing to vehicle communications in recent years [16,17,18,19,20,21,22,23,24]. As a result, node cooperation has become the main consideration, and many techniques to encourage node cooperation were suggested. Several of the existing strategies for vehicle-to-vehicle cooperation tend to focus on Mobile Ad Hoc Network techniques that divide cooperation strategies into four categories: credit-based [25], reputation-based [26,27,28], tit-for-tat based [29], and hybrid-based [30].

The credit-based approach is founded on the notion whereby network nodes can access network resources using a virtual currency; for instance, the node should pay to obtain or use network services, and thus nodes are rewarded for providing or sharing those services with all other network nodes. Reputation-based strategies, on the other hand, are using a tracking method to identify misbehavior nodes. They then send out an alert message across the network to notify all nodes of the existence of these types of nodes. This alert message is used by nodes to prevent or take action toward selfish or misbehaving nodes (e.g., punishing or encouraging them). The tit-for-tat strategies are also the most famous methods since these are very straightforward. Each node throughout these approaches forwards messages to its neighbors in the same way that the neighbors forward messages to it. In hybrid-based techniques, both credit-based and reputation-based strategies are combined to encourage nodes for cooperation within a network. This section summarizes and addresses the major contributions to all cooperative techniques for vehicular networks by separating them into credit-based, reputation-based, tit-for-tat, and hybrid-based strategies. The following are the credit-based strategies used for cooperation.

Chen et al. [31] proposed a secure credit-based approach called the earliest path singular rewarding (EPSR) scheme to encourage selfish and malicious nodes to actively take part in the packet forwarding in non-cooperative DTN. In this scheme, credit is awarded to the selfish and malicious nodes by showing cooperation with all other nodes in the network. Seregina et al. [32] proposed a reward-based incentive strategy to handle the issue of selfishness in DTN. In this strategy, the relay nodes are given payment after successfully delivering packets from the source to the destination. Sharah et al. [33] proposed a credit-based scheme to tackle the problem of selfishness in MANETs. They introduce a slave mode selfish dynamic punishment strategy that uses a cooperative repeated game to prevent selfish conduct in MANET and encourage selfish nodes for cooperation. The approach is used to impose a cooperative punishment on all network nodes to fatigue the punished node and encourage it to collaborate with other participants. SCR is a routing protocol proposed by Haigang et al. [34] for vehicular networks. This routing system is based on the concept of social contribution and is capable of dealing with selfish or misbehaving nodes. SCR incorporates two criteria for making forwarding decisions: delivery likelihood and a network node’s social contributions. The social connection is generated by reciprocal and communal contributions, while the probability of node delivery is estimated, relying on the social affiliations among nodes. The social impact is often considered to encourage selfish nodes to communicate, cooperate and share their resources. Jiang et al. [35] introduce a secure credit-based incentive strategy (SCIS) for single-copy routing in opportunistic networks to deal with the problem of selfishness. The technique is reward compatible and, therefore, can successfully imitate selfish nodes forwarding messages cooperatively. The following are the reputation-based strategies used for cooperation.

Rehman et al. [27] proposed an honesty-based democratic scheme to motivate selfish nodes to cooperate in the internet of things-based vehicular delay-tolerant networks. In the democratic process, different leaders such as cluster head, incentive head, and monitoring head are elected based on two important characteristics such as honesty level and cooperation. These elected heads perform different roles inside the cluster. Loudari et al. [36] proposed a novel reputation mechanism called Distributed Approach for Selfishness Handling (DASH) in a DTN to cope with selfish nodes. Instead of permanently removing the selfish nodes from the network, they seek to prevent communication with them until they collaborate once more. As a result, selfish nodes are given the chance to adjust their behavior, assist in package forwarding and, therefore, enhance the performance of the network. Rehman et al. [28] proposed a socially omitting selfishness (SOS) scheme to handle the issue of selfishness in smart and connected communities in IoT. This scheme uses the extended version of the Dempster–Shafer model to discourage selfish nodes in the communities. When the nodes show selfish behavior repeatedly, such nodes are also penalized in the form of removal from the community. Park et al. [37] presented a long-term reputation system that focuses its effectiveness on regular evaluations of roadside infrastructure. This concept describes car reputation scores by observing approaching vehicles regularly. To achieve this, the approach needs the use of a private and verified credential for vehicles. Dias et al. [38] proposed a reputation system for VDTNs. To distinguish the mobile selfish nodes from the cooperative nodes, this model employs a reputation criterion. A node is characterized as a cooperative node if its reputation score exceeds the reputation criterion; otherwise, it is designated as a selfish node. The following are the tit-for-tat strategies used for cooperation.

Wahab et al. [39] proposed the Dempster–Shafer-based tit-for-tat technique using a QoS-OLSR protocol to deal with the issue of vehicle cooperation in a VANET with selfish nodes. QoS-OLSR is one of the proactive protocols that evaluate the nodes’ Quality of Service (QoS) when electing cluster heads and picking MPR nodes. Cluster heads and MPRs can behave badly on the road. To study the connection between vehicles, traditional and helpful tit-for-tat is introduced. Al-Terri et al. [40] introduced two collaborative-based tit-for-tat approaches called Group Reputation and Cooperative Detection strategies. Both techniques can strengthen the determination to identify misbehavior and therefore improve MAC-layer cooperation in VANETs. The reputation of the node’s neighbors is combined in the Group Reputation tit-for-tat technique, whereas the reputation of the node’s neighbors is grouped in the Cooperative Detection tit-for-tat approach. The following are the hybrid incentive strategies used for cooperation.

Charilas et al. [41] proposed a new hybrid reward system called ICARUS that relies on DARWIN, a popular reputation-based system that combines the benefits of both reputation-based and credit-based mechanisms. ICARUS aims to successfully identify and punish selfish nodes and also motivate nodes to cooperate by encouraging packet forwarding. Moreover, ICARUS guarantees that different nodes are treated equally and that selfish nodes do not compromise the system by providing false information. Wang et al. [42] proposed a reputation-based credit model (RCM). It is a new hybrid reward framework that incorporates payment risk relying on reputation.

In the non-cooperation game, when associated with a routing cost paradigm neighboring nodes gain a Nash equilibrium that strategically offers a reasonable decision on the assignment of routing activities for transmitters within an appropriate incentive.

Different incentive-based schemes have been discussed in the literature. However, no mechanisms for identifying selfish nodes to better understand their impact on the network were taken into consideration. In this article, an Efficient Monitoring System (EMS) is presented to help network nodes identify selfish nodes. To do this, EMS assigns a reputation value to each network node. Therefore, the EMS modifies a node’s reputation value anytime it actively engages in a communication process based on four subsystems (nodes categorization, neighborhood assessment, punishment, and recommendation). Table 1 shows the summary of all the strategies discussed in the related works.

## 3. Problem Statement

The issue of misbehaving and selfish nodes, which leads to network efficiency deterioration when no measures are taken against them, is addressed in this article. A case study was performed that used the NS2 simulation tool [43] to demonstrate the effect of these nodes on the output of VDTNs. A map-based depiction (4000×3200 m${}^{2})$ is included in the simulation. During a 48-hour simulation, these network nodes interact via IEEE 802.11b (at 8 Mbps) and Omni-directional antennas with a communication range of 300 m. Ten terminal nodes, including a buffer capacity of 120 MB, serve as traffic sources and sinks. There were five relay nodes located at five road crossing points to increase the number of network connections. Each relay does have a 120 MB buffer space. A group of 25 to 120 mobile nodes travels across map paths at a speed of 40 km/h with a buffer space of 60 megabytes. The amount of selfish nodes begins at 0% (when there are no selfish nodes) and progressively increases up to 80% of all the nodes throughout the entire simulations (20 for each juncture). No selfish node identification frameworks were taken into account to better explain their effect on the network.

**Figure 1:**
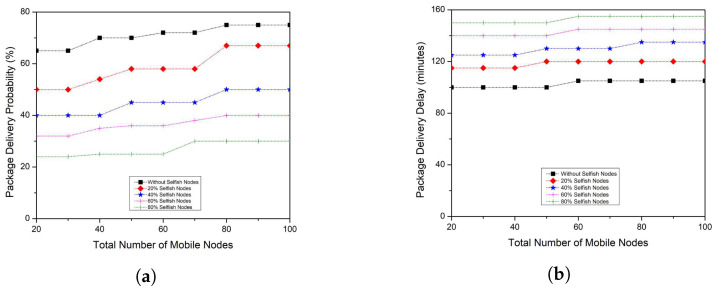

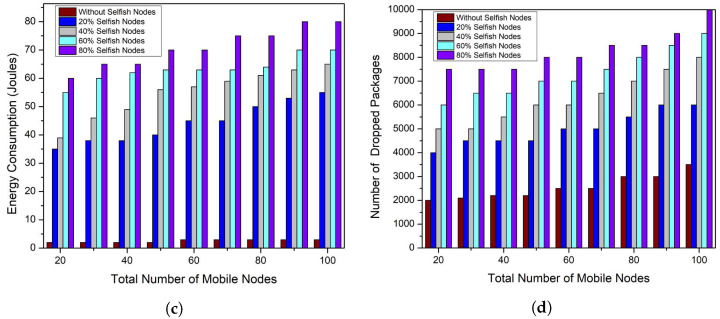
Influence of selfish nodes on all four performance metrics. (**a**) Package Delivery Probability; (**b**) Package Delivery Delay; (**c**) Average Energy Consumption; (**d**) Number of Dropped Packages.

The work begins with the assessment of the effect of selfish nodes on the percentage of bundles distributed that can be seen in Figure 1a. As can be shown, the number of distribution packages gradually decreases when the number of selfish nodes increases rapidly. This activity emphasizes the significance of identifying such nodes so that measures can be taken toward them (e.g., punishing selfish nodes). These selfish nodes affect the time it takes for packages to reach their intended destination. This occurs because selfish nodes compel cooperative nodes to almost double their efforts to produce packages. Cooperative nodes, for instance, will also have to deliver packages for longer periods before delivering them to their intended destination or some cooperative node. The time it takes for a package to move in between the source and recipient nodes would increase dramatically in this process, as can be seen in Figure 1b. Buffer congestion occurs when packages are placed on nodes over long periods, causing a greater ratio of dropped packages because nodes should keep their cooperative activities to not diverge from the underlying protocol. Nodes can, however, drop packages and thus save resources and sustain the integrity of data. The presence of selfish nodes within a network does have a significant effect not just on several nodes but also on routing schemes.

The energy consumption parameter, which represents a routing protocol’s energy efficiency, can be seen in Figure 1c. As can be shown, the presence of selfish nodes within a network increases the routing protocol energy consumption substantially, owing to inadequate network efficiency. The number of packages dropped as a result of the presence of selfish nodes can be seen in Figure 1d. The implications of selfish nodes within the network, as mentioned in this section of the article, could result in a disastrous scenario that can be seen in Figure 1. For this, it is essential to provide nodes with specialized models that can identify and prevent any misbehaving or selfish node. As a result, an EMS for VDTNs is introduced as a solution for coping with the existence of such selfish nodes in the network.

## 4. Proposed Efficient Monitoring System (EMS)

The key characteristics of the EMS developed for VDTNs are presented in this section. The major purpose of the EMS is to provide VDTN nodes in the network with an efficient approach for detecting nodes that deviate from the protocol.

### 4.1. Basic Concepts

Every network node in the EMS does have a reputation value (γ) that can be used to calculate the number of resources that nodes can exchange with the other nodes in the network (for instance, buffer needed to keep packages from others or communication time spent forwarding packages from others). At first, this value is 45; however, that may vary between 0 and 100 over time. The nodes in EMS can contact one another.

**Figure 2:**
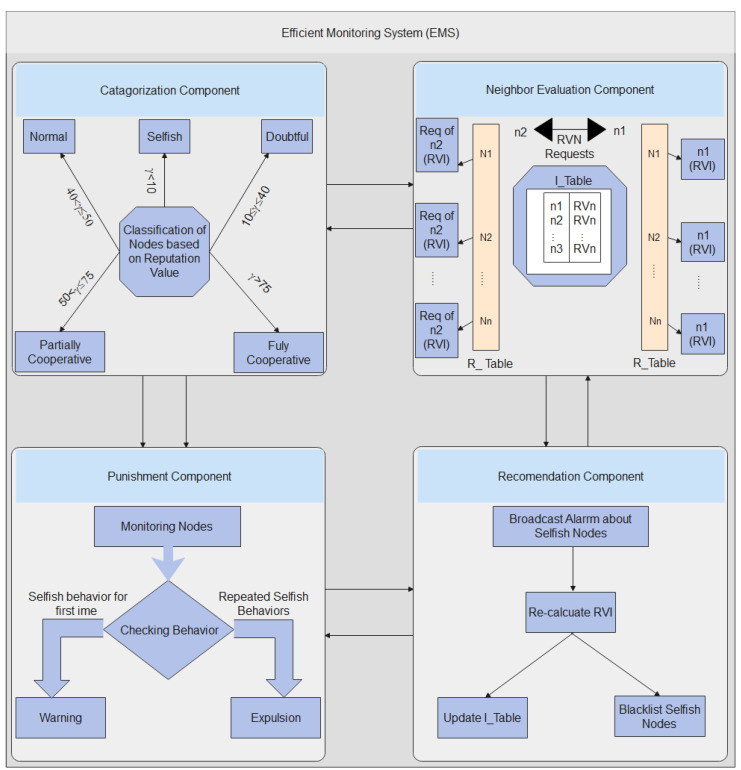
Overall Structure of Proposed Scheme.

Nodes share information regarding their system efficiency (e.g., number of relayed, dropped, and forwarded packages) at each encounter, allowing nodes to analyze one another. Such information is often received by EMS, which will preserve data on every network node’s performance. The EMS thereafter changes each cooperating node’s reputation value based on three separate values at each interaction opportunity. A cooperative value is given by the monitoring node (CVM), a node reputation value viewed by neighbors (RVN), as well as a node reputation value noticed by the node on its own (RVI). The EMS comprises four main components to produce these values: a node categorization component, a neighborhood assessment subsystem, a punishment component, and a determination subsystem. The overall structure of the proposed scheme is shown in Figure 2. The notations used in this article are shown in Table 2.

### 4.2. The Node Categorization Component

The categorization component’s major purpose is to categorize nodes based on their effect on the entire network performance. This component handles a categorization table that stores a record of every network node and is refreshed after each encounter opportunity to accomplish this task. Each record does have a unique ID that specifies a node, as well as the most recent reputation value computed by EMS and its cooperative value (CVM). The cooperative value of a node *n* is obtained using Equation (1).

(1) $$CVM_{i}=\eta \times \Delta i$$

where η is the node efficiency factor and Δ denotes the punctuation assigned to node *i* by the categorization unit. A node efficiency factor is a number that indicates how well each node performs inside the network. The categorization component employs Equations (2) and (3) to determine this value, whereas $TRP_{m}$ is the total number of relayed packages from node *m*, $TFP_{m}$ is the total number of packages that node *m* has recently forwarded, and $TDP_{m}$ is the number of packages that node *m* has previously discarded.

(2) $$y=\Sigma_{m=1}^{N}\left(TRP_{m}-TFP_{m}\right)$$

(3) $$z=\Sigma_{m=1}^{N}\left(TRP_{m}-TDP_{m}\right)$$

Taking into account Equations (4) and (5), this value is adjusted to range around [0, 1].

(4) $$\eta_{1}=\frac{y-y_{min}}{y_{max}-y_{min}}$$

(5) $$\eta_{2}=\frac{z-z_{min}}{z_{max}-z_{min}}$$

The categorization component calculates the punctuation assigned to nodes Δ depending on their classification. Based on their reputation value, nodes can be categorized into five types. To compute Δ, presume Equation (6), where *j* is a fixed value derived from the monitoring of a node’s reputation value, which can be seen in Table 3, and ω is a constant established by the EMS as the criterion for rewarding or penalizing nodes based on their cooperative attitude.

(6) $$\Delta_{i}=j\times \omega$$

**Figure 3:**
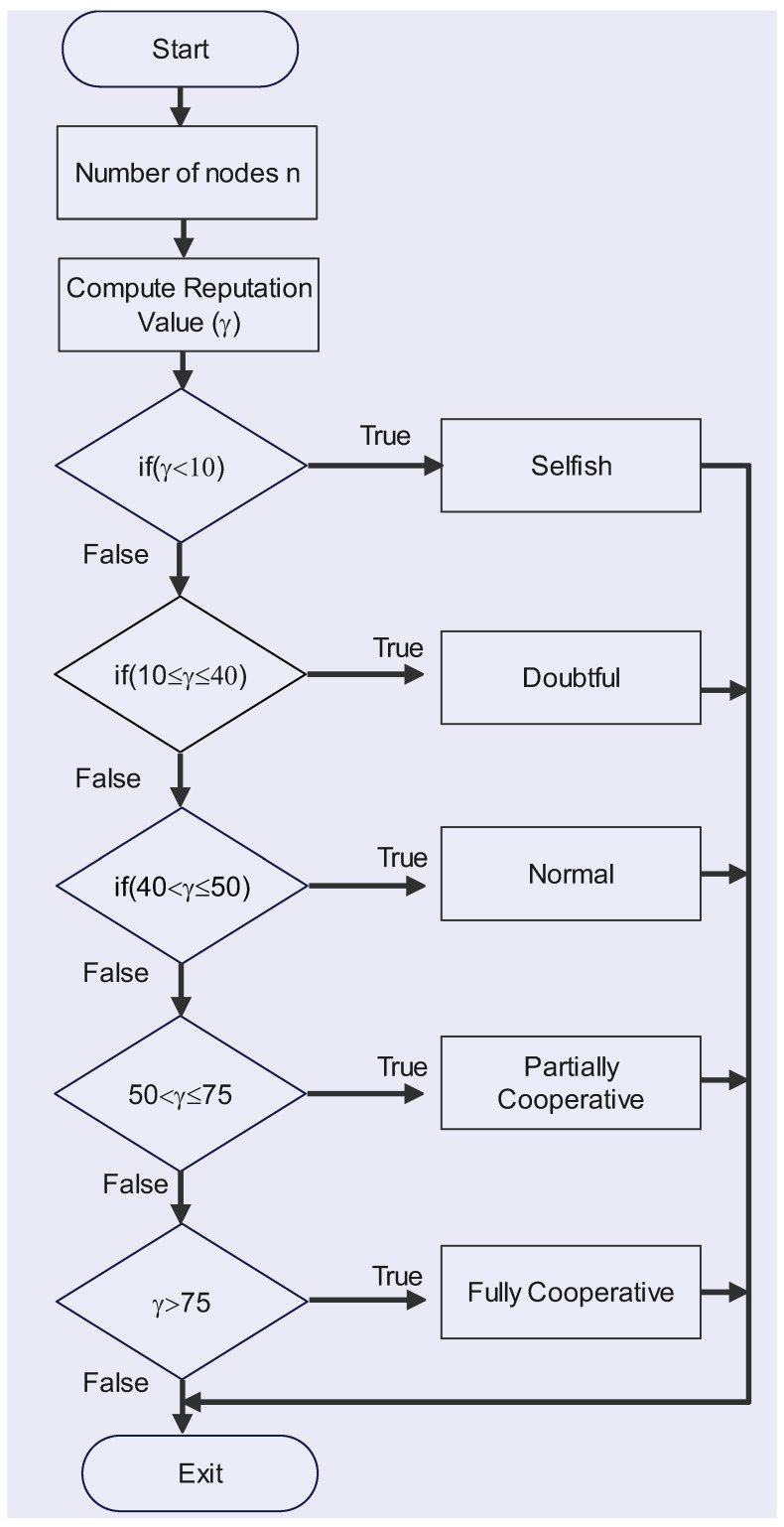
Flowchart of the Categorization Component.

The detail of the categorization component is presented in Algorithm 1. In this algorithm, the first monitoring node determines the CVM of each node in the network. The CVM is based on two things: (1) relayed packages for nodes (2) discarding packages for nodes. After this, the reputation of each node is computed. Based on the reputation value, the node can be classified into one of the five classes. The flow chart for the categorization component is shown in Figure 3.
**Algorithm 1** Algorithm for Categorization Component.  **INPUT:** Number of Nodes *n*  **OUTPUT:** Nodes Classification1:**for** i=1:n **do**2:    Compute CVM of node3:    $CVM_{i}=\eta \times \Delta i$4:    **if** Node relayed packages for a node *m* **then**5:        $y=\Sigma_{m=1}^{N}\left(TRP_{m}-TFP_{m}\right)$6:        $\eta_{1}=\frac{y-y_{min}}{y_{max}-y_{min}}$7:    **else**8:        **if** Node discard packages for a node *m* **then**9:           $z=\Sigma_{m=1}^{N}\left(TRP_{m}-TDP_{m}\right)$10:           $\eta_{2}=\frac{z-z_{min}}{z_{max}-z_{min}}$11:        **end if**12:    **end if**13:**end for**14:**for all** j∈n **do**15:    Compute reputation value γ of each node16:    **if** γ<10 **then**17:        Node ==’Selfish’18:    **else**19:        **if** 10≤γ≤40 **then**20:           Node==’Doubtful’21:        **else**22:           **if** 40<γ≤50 **then**23:               Node==’Normal’24:           **else**25:               **if** 50<γ≤75 **then**26:                   Node==’Partially Cooperative’27:               **else**28:                   Node==’Fully Cooperative’29:               **end if**30:           **end if**31:        **end if**32:    **end if**33:**end for**34:End

### 4.3. Neighbor’s Assessment Component

This component’s primary goal is to share reputations with immediate neighbors. The RVN value of each node is computed by the neighbor assessment component. Equation (7) can be used to compute the RVN value. This component of EMS comprises two types of tables; namely, information table $I_{Table}$ and references table $R_{Table}$. Each entry in $I_{Table}$ comprises a node ID and the RVN value for that node. This component uses three different types of messages to share reputations with its neighbors, namely $Reputation_{request}$, $Reputation_{reply}$ message, and $ALARM_{message}$. The neighbor’s assessment component asks *N* neighbors to offer their comments on the member nodes at each communication opportunity through $Reputation_{request}$ message. The RVN value for the node responding to the neighbor’s assessment component query is returned by these neighbors through $Reputation_{reply}$ message. These values are saved in the $R_{T}able$, which each neighbor is responsible for maintaining throughout its time on the network. The $ALARM_{message}$ message is sent to neighbors regarding a node who performs selfish behavior repeatedly and is currently punished. Each time a neighbor makes direct communication with a node *i*, the RVN value of a such node is updated.

(7) $$RVN_{i}=\frac{\Sigma_{n=1}^{N}R_{v}}{N}$$

The detail of the neighbor’s module is discussed in Algorithm 2. In this algorithm, nodes send requests to obtain the reputation of certain nodes from their neighbor’s nodes. The neighbor node checks the reputation of nodes in $Ref_{T}able$ and its view on the reputation of those specific nodes. After sending $reply_{m}essage$, the neighbor’s node updates the $R_{T}able$.
**Algorithm 2** Algorithm for Neighbor’s Assessment Component  **INPUT:** *N* neighbor’s to determine node *i*  **OUTPUT:** RVN of node *i*1:view = 0;2:node send $Reputation_{request}$ to its neighbor’s3:**for** each node i∈N **do**4:    $veiw=view+node_{i}\left(RVN\right)$5:    $RVN=\frac{view}{N}$6:    Neighbor’s send back $Reputation_{reply}$ to node asked reputation of $node_{i}$7:    update $R_{T}able$8:    update $I_{T}able$9:**end for**10:End

### 4.4. Punishment Component

**Figure 4:**
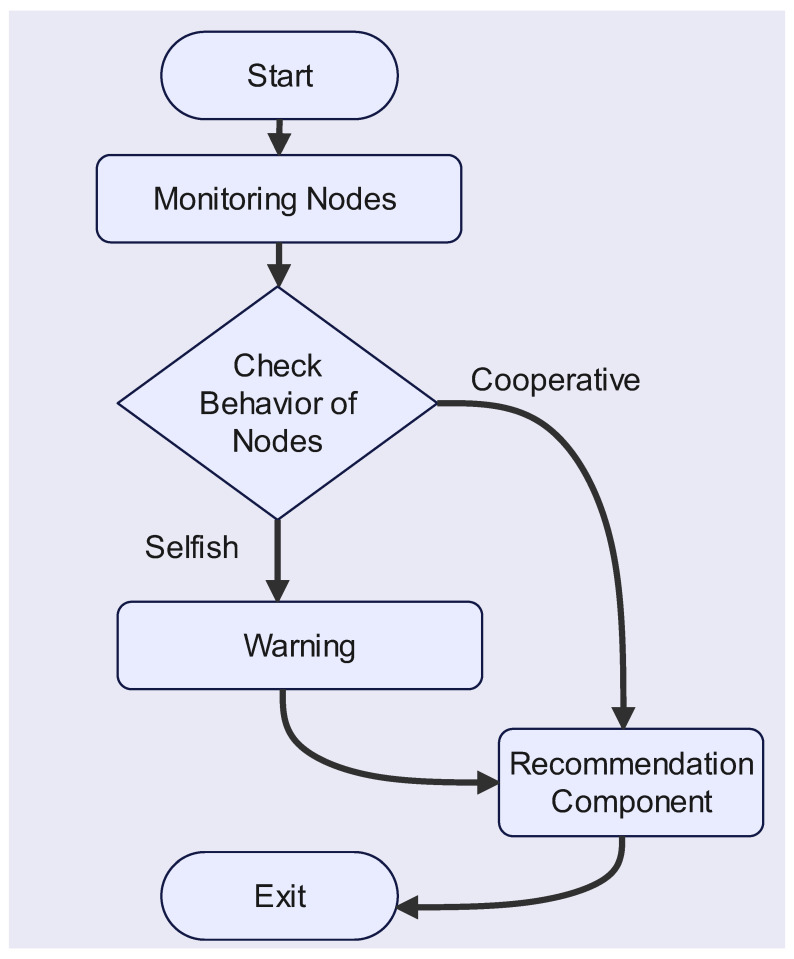
Flow Chart of the Punishment Component.

In this component of the proposed scheme, the behavior of the nodes is constantly checked by the monitoring nodes. If the behavior of the nodes is found to be selfish for the first time, it warned such nodes in the network. However, when selfish nodes behave selfishly repeatedly, then nodes are punished in the form of exclusion from the network and their reputation value is decreased. The punishment given to selfish nodes can be calculated by using Equation (8).

(8) $$PN_{i}=\lambda_{1}ON_{i}+\lambda_{2}O_{i}$$

where $PN_{i}$ is the punishment to node *i*, $ON_{i}$ is the neighboring node that can verify the behavior of node *i*, and $O_{i}$ is the observation on selfish node *i*. $\lambda_{1}$ and $\lambda_{2}$ are weight variables, and $\lambda_{1}$ + $\lambda_{2}$ = 1. These weight variables could be included to support and create flexibility only for the node punishment feature, where $\lambda_{1}$ helps to measure the supporting variable appropriate for a node’s number of neighbors and $\lambda_{2}$ gives a weight value for the set of observations created by neighbors. A selfish node is identified as one with a reputation value of less than 10. This sends out a warning to all the neighbors of the node, allowing this to propagate throughout the network. Collecting this warning message indicates that a node has been labeled as selfish and should be submitted to a node’s blacklist. The network ignores and discards nodes on the blacklist. The flow chart of the punishment component is shown in Figure 4.

### 4.5. Recommendation Component

The recommendation component considers the information provided by the categorization component CVM, the neighbor’s assessment system RVN, and the reputation value obtained by the node of its own to change the reputation value γ of a network node only at the end of a communication period RVI. The RVI is obtained through an interface that interacts with each network node’s VDTN reputation mechanism. The nodes may generate a view of their outcomes using this reputation method. The truly new node’s reputation score (γi) is determined by adding all three values together, as shown in Equation (9).

(9) $$\gamma i=\psi RVI_{i}+\left(1-\psi \right)RVN_{i}+CVMi$$

where ψ is a number within a range [0, 1] that indicates how much the EMS believes the node’s findings. The recommendation component sends all nodes’ reputation values to the categorization component that updates its categorization table until recalculating nodes γ. Nodes are also notified of their new reputation values by the EMS. Table 4 shows the comparison of the proposed system with other schemes.

## 5. Performance Evaluation

The proposed Efficient Monitoring System has been thoroughly evaluated, and its efficiency has been examined using NS-2. The NS-2 is only a discrete event-driven network simulation tool for studying how dynamic communication networks exist. Regarding simulating various protocols via wired and wireless networks, NS-2 offers comprehensive support. With support for various network components, protocols, traffic patterns, and routing types, it offers a highly adaptable framework for wired and wireless simulations. The major goal of this work is to see how effective the suggested monitoring is in detecting selfish nodes inside the network and how it helps the entire network improve in performance. The Incentive and Punishment Scheme (IPS) [26] and Socially Omitting Selfishness (SOS) [28] are used as a benchmark in this work. The performance metrics considered for simulations are package delivery probability, delivery delay, energy consumption, and the number of packet drops. Table 5 shows all the performance parameters used in the simulation.

The EMS was implemented in similar system parameters as in the Section 3 test case. Whenever the EMS is compared to a situation where there are no selfish nodes, and monitoring is done under similar circumstances, the overall network performance can be compared.

### 5.1. Impact of Selfish Nodes on All Performance Metrics

**Figure 5:**
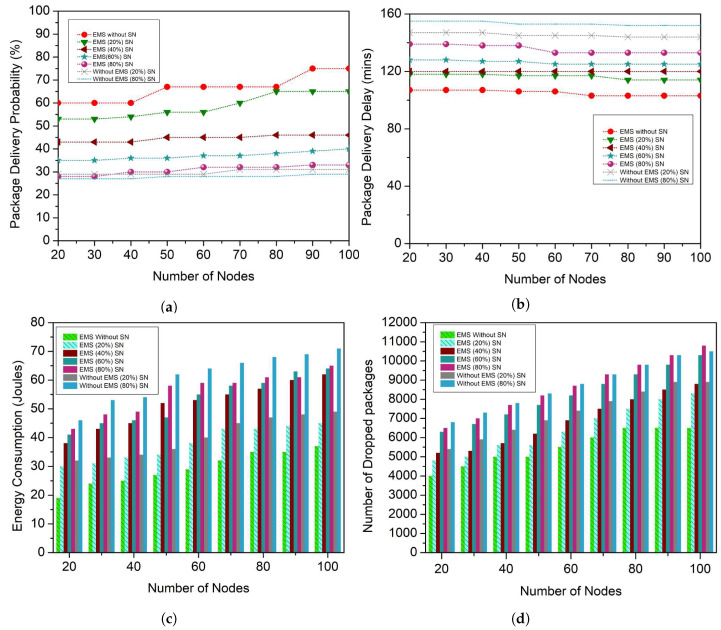
Impact of a Varying Number of Selfish Nodes. (**a**) Package Delivery Probability; (**b**) Package Delivery Delay; (**c**) Energy Consumption; (**d**) Number of Dropped Packages.

The observed results of the EMS technique were compared to techniques that did not undertake selfish node detection to determine the performance of the proposed EMS, as shown in Figure 1. The percentage of delivered packages is the first step in this investigation. As seen in Figure 5a, the package delivery probability decreases as the number of selfish nodes grow. The EMS, on the other hand, attempts to mitigate the negative effects of selfish nodes. This can be verified by comparing the package delivery probability of EMS with those methods that take no measures towards selfish nodes. When analyzing both techniques when 20% of nodes behave selfishly, as can be seen in Figure 1, the EMS enhances package delivery probability by about 28%, 23%, 25%, 27%, 28%, 26%, 34%, 32%, and 33% (for 20, 30, 40, 50, 60, 70, 80, 90, 100 mobile nodes, respectively).

Moreover, in the worst-case sort of situation (80% selfish nodes), EMS helps to minimize the effect of selfish nodes, which will increase package delivery probability by 12%, 12%, 14%, 13%, 13%, 13%, 13%, 12%, and 12% (for 20, 30, 40, 50, 60, 70, 80, 90, 100 mobile nodes, respectively), particularly in comparison to a method that takes no action against selfish nodes. EMS is not just useful when there are selfish nodes in the network. When compared and contrasted to a strategy where no reward is offered to cooperative nodes (i.e., nodes that do not deviate from the policy), the suggested cooperative scheme also helps to enhance the package delivery probability that can be seen in Figure 1a. The EMS then improves the package delivery probability by roughly 14%, 15%, 15%, 16%, 16%, 16%, 15%, 15%, and 16% (for a number of mobile nodes of 20, 30, 40, 50, 60, 70, 80, 90, 100, respectively). This is because, in the EMS scheme, there are *N* nodes to exchange their views on other nodes’ routing behavior with one another, allowing them to identify a substantial percentage of selfish nodes.

When compared to a technique for which no selfish node identification is conducted, which can be seen in Figure 1b, the EMS also obtains good results in terms of the package average delivery delay, as shown in Figure 5b. It indicates that EMS can send packages faster, which is much more obvious when the network has 60 or more mobile nodes. When 20% of selfish nodes are taken into account, the EMS delivers packages usually 22, 22, 22, 21, 21, and 21 min earlier (for 50, 60, 70, 80, 90, and 100 mobile nodes, respectively). Furthermore, in the worst-case situation (80% selfish nodes), the EMS handles packages that arrive 15, 15, 18, 18, 18, and 21 min earlier. It takes a very long time for a package to reach the destination node when the amount of selfish nodes grows in the network. When there is a selfish node in the network, packages will be discarded or significantly delayed, forcing the network to re-transmit the data packets. Re-sending data packets wastes network power, shortens the lifetime of the network and increases packet delivery delay. The package delivery delay is minimized in EMS because selfish nodes are detected quickly.

The effectiveness of the EMS is examined in terms of energy consumption and package drop rate. For such a reason, the energy is taken into account first. The energy consumption does not vary considerably as a result of the EMS strategy, as shown in Figure 5c, despite the rise in the number of selfish nodes. When compared to the results obtained when no measures are taken over selfish nodes, as shown in Figure 1c, this is a substantial improvement. The EMS reduces the energy consumption by nearly 6, 7, 7, 8, 8, 8, 8, 9, and 9 packages (20, 30, 40, 50, 60, 70, 80, 90, and 100 mobile nodes, respectively) in the worst-case situation (80% of nodes behaving selfishly). This occurs as a result of EMS rewarding nodes for their cooperation, encouraging cooperative nodes to exchange much more resources. In terms of dropped packages, as seen in Figure 5d, the EMS helps to reduce dropped packages for all the other examined selfish node percentages. Particularly, in comparison to a similar strategy without any selfish node detection, the EMS drops 200, 200, 250, 250, 250, 260, 260, 265, and 265 bundles for the 20% strategy, as shown in Figure 1d. The EMS can discard fewer than 500, 512, 500, 560, 566, 567, 567, 570, and 570 packages in the worst-case situation. By monitoring the existence of selfish nodes and minimizing their interaction with cooperative nodes, the EMS can reduce network resources (such as buffer storage space). This saves resources for cooperative nodes that only use them to forward packages to certain other cooperative nodes. It is also due to the reason that many packets are transferred among the nodes; whenever the number of selfish nodes inside the network is significant, more energy is required to identify selfish nodes. The suggested method can identify selfish nodes in shorter durations, resulting in fewer packet drops and less energy consumed when re-sending packets via the network.

### 5.2. Comparison of EMS, IPS and SOS Schemes for Selfish Nodes of 20% to 80%

**Figure 6:**
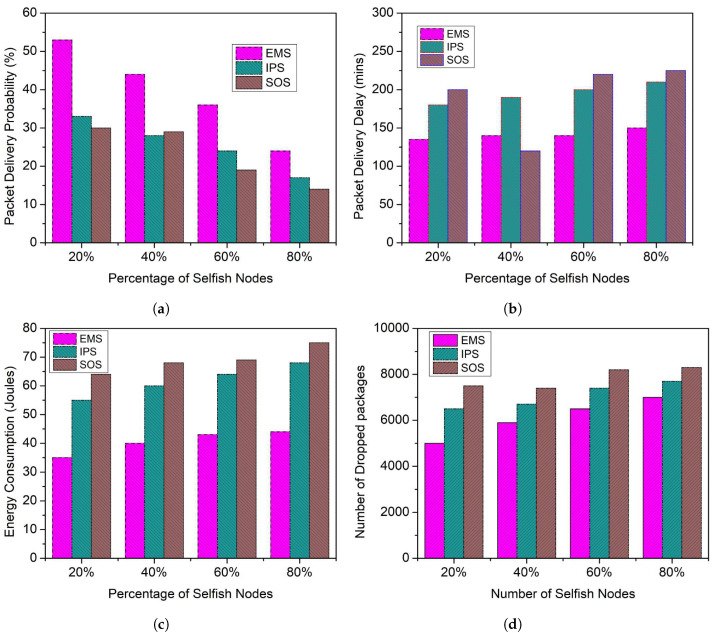
Comparison of EMS, IPS, and SOS schemes for Selfish Nodes of 20% to 80%. (**a**) Packet Delivery Probability; (**b**) Packet Delivery Delay; (**c**) Energy Consumption; (**d**) Number of Dropped Packages.

In this section, the performance of the proposed system is compared with the existing scheme, namely IPS and SOS, for different percentages of selfish nodes. In the first case, 20% of selfish nodes are taken. In this case, the packet delivery probability of the proposed scheme EMS, IPS, and SOS are 52%, 33%, and 29%, respectively, 31% and 37% higher than IPS and SOS, as seen in the Figure 6a. In another case, 80% of selfish nodes are taken. Here, the packet delivery probability of EMS, IPS, and SOS is 24%, 19%, and 15%, which is still 9% and 15% better than IPS and SOS.

As shown in Figure 6b–d, the packet delivery delay, energy consumption, and the number of packages dropped in the Proposed EMS scheme is lower. For 20% selfish nodes, the packet delivery delay of EMS, IPS, and SOS is 120, 127, and 190 min, which is 2% and 23% lower than IPS and SOS. While taking 80% selfish nodes, the packet delivery delay of the EMS, IPS, and SOS is 160, 215, and 225 min, respectively, which is 18% and 22% lower than IPS and SOS. In Figure 6c, the energy consumption of the EMS, IPS, and SOS schemes is 35, 55, and 65 joules, respectively, which is 23% and 37% lower than IPS and SOS when 20% of nodes are behaving selfishly in the network. Similarly, for 80% selfish nodes, the energy consumption of the EMS, IPS, and SOS schemes is 45, 65, and 75 joules, respectively, which is 25% and 37% lower than IPS and SOS. In addition, for 20% of nodes behaving selfishly in the network, the number of dropped packages for the EMS, IPS, and SOS schemes is 4500, 6200, and 7800, respectively, as shown in Figure 6d, which is 17% and 33% lower than IPS and SOS. Similarly, for 80% selfish nodes, the number of dropped packages of the EMS, IPS, and SOS schemes is 6500, 6700, and 8100, respectively, which is 2% and 16% lower than IPS and SOS. The main reason for this is that the EMS approach enables selfish nodes to partake in packet forwarding, hence preventing selfish behavior. The other two strategies did not go into detail about how selfishness affects the network. Furthermore, the nodes show cooperativeness in the network due to the fear of punishment. Furthermore, the findings demonstrate that the EMS scheme can accurately handle a huge variety of selfish nodes by permitting them to collaborate in a network to improve network performance.

## 6. Conclusions and Future Work

To cope with the existence of selfish or non-cooperative nodes in a network, this article developed an EMS for VDTNs. This type of node has a significant impact on the entire network efficiency and may compromise cooperative node effectiveness because they consume resources (such as energy, memory, and buffer) from other network nodes to meet their demands. Such nodes are regarded as nodes having reputation values less than 10 as they decline packets immediately after reception. The EMS depends on the cooperative transfer of values used for nodes’ reputation, as well as the four modules (categorization, neighbor assessment, punishment, and recommendation) to find and eliminate the selfish nodes from the entire network.

The results of the proposed EMS are conducted in the NS2 simulation tool, which shows that EMS is effective at reducing the impact of selfish nodes on the performance of the entire network. The EMS is compared with two other existing schemes, namely IPS and SOS. The EMS surpasses the benchmark schemes in terms of packet delivery probability, package delivery delay, energy consumption, and the number of dropped packages. We plan to expand this monitoring system for energy-constrained selfish VDTNs in the future.

## Figures and Tables

**Table 1:** Summary of all the strategies used in related works.

Article & Authors	Contributions	Disadvantages	Advantages
Rehman et al. [26]	Proposed an Incentive and Punishment Scheme (IPS) to deal with the problem of selfishness in the Internet of Vehicles (IoV)	Whenever the number of selfish nodes increases, its performance degrades	A proper solution for a weight-tie problem
Rehman et al. [28]	Proposed a Socially Omitting Selfishness (SOS) scheme to handle the issue of selfishness	No categorization of selfish nodes	It can be used for both intra and inter-communities
Chen et al. [31]	Proposed a secure credit-based approach called the earliest path singular rewarding (EPSR) scheme to encourage selfish nodes for cooperation	No incentive differentiation (fixed incentive to all nodes)	The reputation score of a node is increased only when it successfully delivered the bundles
Seregina et al. [32]	Proposed a reward-based incentive strategy to handle the issue of selfishness	Performance degrades when selfish nodes in the network increases	The relay nodes are given payment after successful delivery
Sharah et al. [33]	Credit-Based scheme to tackle the problem of selfishness	Strict Punishment (node once punished cannot join the network again)	Warning node for showing selfish behavior for the first time
Haigang et al. [34]	Proposed a routing scheme called SCR to handle the issue of selfishness	Nodes with higher threshold reputations may be expelled as they are not qualified	Nodes are encouraged to cooperate socially
Jiang et al. [35]	Introduce a Secure Credit-Based Incentive Strategy (SCIS) for single-copy routing in opportunistic	Individual selfishness (nodes do not share their resources with anyone)	Social selfishness (nodes share their resources with all other nodes to whom it has a social relationship )
Loudari et al. [36]	Proposed a novel reputation mechanism called Distributed Approach for Selfishness Handling (DASH) in a DTN	Ignore selfish behaviors such as manipulation and self-centeredness, etc.	Selfish node detection message is broadcasted to all other nodes in the network
Park et al. [37]	Presented a long-term reputation system for selfishness	It does not determine the collusion among nodes	Message encryption
Dias et al. [38]	Proposed a reputation system to deal with the issue of selfishness in VDTNs	Sometimes watchdog nodes can also be selfish	Watchdog nodes constantly check the behavior of all other nodes
Wahab et al. [39]	Proposed the dempster–Shafer based tit-for-tat technique using QoS-OLSR protocol	High overhead	Encourage truth-telling using Dempster–Shafer Model
Al-Terri et al. [40]	Introduced two collaborative-based tit-for-tat approaches called Group Reputation and Cooperative Detection	Nothing for omitting selfishness	Improve MAC-layer cooperation in VANETs
Charilas et al. [41]	Proposed a hybrid reward system called ICARUS	Fixed incentive	Nodes are treated equally
Wang et al. [42]	Proposed a reputation-based credit model (RCM) for Selfishness	Each node needed its memory to maintain the reputation file	Encouraged more nodes for cooperation

**Table 2:** Notations used in the Proposed Scheme.

Notations	Description
CVM	Cooperative value given by monitoring node
RVN	Node reputation viewed by neighbors
RVI	Own reputation value of a node
η	Node efficiency factor
Δ	Punctuation assigned to node *i* by the categorization unit.
$\lambda_{1}$	weight variable
$\lambda_{2}$	weight variable
ψ	Node findings in the range [0, 1]
*N*	Neighbor node
$R_{v}$	Neighbor view on node reputation
γ	Node reputation viewed by neighbors
$ON_{i}$	Neighboring nodes that can verify the behavior of node *i*
$O_{i}$	is the observation on selfish node *i*
$PN_{i}$	Punishment to node *i*
ω	Constant established by the EMS (rewarding or penalizing nodes)
$TRP_{m}$	Total number of relayed packages
$TFP_{m}$	Total number of packages that node *m* has recently forwarded
$TDP_{m}$	Number of packages that node *m* has previously discarded.

**Table 3:** Value of constant *j* according to classification of node in EMS.

Value of *j*	Type of Node	Reputation Value (γ)
−1.0	Selfish	γ<10
−0.5	Doubtful	10≤γ≤40
0.0	Normal	40<γ≤50
0.5	Partially Cooperative	50<γ≤75
1.0	Fully Cooperative	γ>75

**Table 4:** Comparison of the proposed system with other schemes.

Article & Authors	Contributions	Weaknesses	Comparison with EMS
Chen et al. [31]	Proposed a secure credit-based approach called the earliest path singular rewarding (EPSR) scheme to encourage selfish nodes for cooperation	No incentive differentiation (fixed incentive to all nodes)	Variable incentives (depends on the degree of cooperation)
Sharah et al. [33]	Credit-based scheme to tackle the problem of selfishness	Strict punishment (Node once punished can not join the network again)	Warn node for showing selfish behavior for the first time
Jiang et al. [35]	Introduce a Secure Credit-Based Incentive Strategy (SCIS) for single-copy routing in opportunistic networks to deal with the problem of selfishness	Individual selfishness (nodes do not share their resources with anyone)	Social selfishness (nodes share their resources with all other nodes to whom it has a social relationship )
Rehman et al. [28]	proposed a Socially Omitting Selfishness (SOS) scheme to handle the issue of selfishness in smart and connected communities in IoT	No categorization of selfish nodes	Selfish nodes are properly categorized
Dias et al. [38]	Proposed a reputation system to deal with the issue of selfishness in VDTNs	Sometimes watchdog nodes can also be selfish	Monitoring nodes are also properly monitored by the other nodes in the network
Rehman et al. [26]	Proposed an Incentive and Punishment Scheme (IPS) to deal with the problem of selfishness in the Internet of Vehicles (IoV)	Whenever the number of selfish nodes increases, its performance degrades	It performs better in the case of increasing selfish nodes in the network

**Table 5:** Simulation parameters.

Parameters	Values
Simulation Area	4000×3200 m${}^{2}$
Node Communication	IEEE 802.11b
Transmission Range	300 m
Number of nodes	120
Number of Relay Nodes	05
Number of Terminal Nodes	10
Comparison	Proposed System compared with IPS and SOS Schemes
Simulation Time	48 h
Average Speed	40 km/h
Malicious Activity	0% to 80%
Terminal Nodes Buffer Capacity	120 MB
Relay Node Buffer Capacity	120 MB
Variation in Nodes	20 to 100
Size of Package	[50, 650] KB
Package TTL	320 min
Interval for Package Generation	[20, 30] s
Traffic Source	CBR
Packet Protocol	TCP

## Data Availability

This manuscript has no associated data.

## References

[1] Ashton K. (2009). That ‘internet of things’ thing. RFID J..

[2] Al-Shareeda M.A., Anbar M., Hasbullah I.H., Manickam S. (2020). Survey of authentication and privacy schemes in vehicular ad hoc networks. IEEE Sens. J..

[3] Al-Shareeda M.A., Manickam S., Mohammed B.A., Al-Mekhlafi Z.G., Qtaish A., Alzahrani A.J., Alshammari G., Sallam A.A., Almekhlafi K. (2022). Provably secure with efficient data sharing scheme for fifth-generation (5G)-enabled vehicular networks without road-side unit (RSU). Sustainability.

[4] Cicioğlu M., Çalhan A. (2020). IoT-based wireless body area networks for disaster cases. Int. J. Commun. Syst..

[5] Ma M., He D., Wang H., Kumar N., Choo K.K.R. (2019). An efficient and provably secure authenticated key agreement protocol for fog-based vehicular ad-hoc networks. IEEE Internet Things J..

[6] Mao Y., Zhou C., Ling Y., Lloret J. (2019). An optimized probabilistic delay tolerant network (DTN) routing protocol based on scheduling mechanism for internet of things (IoT). Sensors.

[7] Moetesum M., Hadi F., Imran M., Minhas A.A., Vasilakos A.V. (2016). An adaptive and efficient buffer management scheme for resource-constrained delay tolerant networks. Wirel. Netw..

[8] Kang H., Ahmed S.H., Kim D., Chung Y.S. (2015). Routing protocols for vehicular delay tolerant networks: A survey. Int. J. Distrib. Sens. Netw..

[9] Singh A.K., Pamula R. (2021). An efficient and intelligent routing strategy for vehicular delay tolerant networks. Wirel. Netw..

[10] Rehman G.U., Ghani A., Muhammad S., Singh M., Singh D. (2020). Selfishness in Vehicular Delay-Tolerant Networks: A Review. Sensors.

[11] Dong Y., Zhang F., Lin J.F., Jiao W., Zhang Y. (2020). Learning for Multiple-Relay Selection in a Vehicular Delay Tolerant Network. IEEE Access 8.

[12] Sharma R., Gupta D.V. (2018). A Reputation-Based Mechanism to Detect Selfish Nodes in DTNs. International Conference on Communications and Cyber Physical Engineering.

[13] Mendiboure L., Chalouf M.A., Krief F. (2020). Survey on blockchain-based applications in internet of vehicles. Comput. Electr. Eng..

[14] Zheng H., Xiong K., Fan P., Zhong Z., Letaief K.B. (2021). Age of Information-Based Wireless Powered Communication Networks With Selfish Charging Nodes. IEEE J. Sel. Areas Commun..

[15] Socievole A., Caputo A., De Rango F., Fazio P. (2019). Routing in mobile opportunistic social networks with selfish nodes. Wirel. Commun. Mob. Comput..

[16] Hassija V., Chamola V., Han G., Rodrigues J.J., Guizani M. (2020). Dagiov: A framework for vehicle to vehicle communication using directed acyclic graph and game theory. IEEE Trans. Veh. Technol..

[17] Zhang H., Lu X. (2020). Vehicle communication network in intelligent transportation system based on internet of things. Comput. Commun..

[18] Ou H., Tang T.Q. (2018). An extended two-lane car-following model accounting for inter-vehicle communication. Phys. A Stat. Mech. Appl..

[19] Abbasi I.A., Shahid K.A. (2018). A review of vehicle to vehicle communication protocols for VANETs in the urban environment. Future Internet.

[20] Nguyen D.H.P., Zoltán R. The current security challenges of vehicle communication in the future transportation system. Proceedings of the IEEE 16th International Symposium on Intelligent Systems and Informatics (SISY).

[21] Nakazawa T., Tang S., Obana S. (2020). CCN-based inter-vehicle communication for efficient collection of road and traffic information. Electronics.

[22] Khowaja S.A., Khuwaja P., Dev K., Lee I.H., Khan W., Wang W., Qureshi N.M.F., Magarini M. (2022). A secure data sharing scheme in Community Segmented Vehicular Social Networks for 6G. IEEE Trans. Ind. Inform..

[23] Khan W.U., Jamshed M.A., Lagunas E., Chatzinotas S., Li X., Ottersten B. (2022). Energy efficiency optimization for backscatter enhanced NOMA cooperative V2X communications under imperfect CSI. IEEE Trans. Intell. Transp. Syst..

[24] Khan W.U., Ihsan A., Nguyen T.N., Ali Z., Javed M.A. (2022). NOMA-enabled backscatter communications for green transportation in automotive-industry 5.0. IEEE Trans. Ind. Inform..

[25] Chakrabarti C., Basu S. A blockchain based incentive scheme for post disaster opportunistic communication over DTN. Proceedings of the 20th International Conference on Distributed Computing and Networking.

[26] Rehman G.U., Ghani A., Zubair M., Naqvi S.H.A., Singh D., Muhammad S. (2019). IPS: Incentive and punishment scheme for omitting selfishness in the internet of vehicles (iov). IEEE Access.

[27] Rehman G.U., Ghani A., Zubair M., Ghayyure S.A., Muhammad S. (2021). Honesty based democratic scheme to improve community cooperation for Internet of Things based vehicular delay tolerant networks. Trans. Emerg. Telecommun. Technol..

[28] Rehman G.U., Ghani A., Zubair M., Saeed M.I., Singh D. (2020). SOS: Socially omitting selfishness in IoT for smart and connected communities. Int. J. Commun. Syst..

[29] Mostefa F.Z., Maaza Z.M., Duvallet C. (2020). Secure Communications by Tit-for-Tat Strategy in Vehicular Networks. Int. J. Networked Distrib. Comput..

[30] Park Y., Sur C., Rhee K.H. (2018). A secure incentive scheme for vehicular delay tolerant networks using cryptocurrency. Secur. Commun. Netw..

[31] Chen H., Lou W., Wang Z., Wang Q. (2015). A secure credit-based incentive mechanism for message forwarding in non-cooperative DTNs. IEEE Trans. Veh. Technol..

[32] Seregina T., Brun O., El-Azouzi R., Prabhu B.J. (2016). On the design of a reward-based incentive mechanism for delay tolerant networks. IEEE Trans. Mob. Comput..

[33] Sharah A., Alhaj M., Hassan M. (2020). Selfish dynamic punishment scheme: Misbehavior detection in MANETs using cooperative repeated game. IJCSNS.

[34] Gong H., Yu L., Zhang X. (2014). Social contribution-based routing protocol for vehicular network with selfish nodes. Int. J. Distrib. Sens. Netw..

[35] Jiang Q., Men C., Yu H., Cheng X. A secure credit-based incentive scheme for opportunistic networks. Proceedings of the 7th IEEE International Conference on Intelligent Human-Machine Systems and Cybernetics.

[36] Loudari S., Abouhassane A., Benamar N., Younis M. DASH: A Distributed Approach for Selfishness Handling in a DTN. Proceedings of the 2019 2nd IEEE Middle East and North Africa COMMunications Conference (MENACOMM).

[37] Park S., Aslam B., Zou C. Long-term reputation system for vehicular networking based on vehicle’s daily commute routine. Proceedings of the 2011 IEEE Consumer Communications and Networking Conference (CCNC).

[38] Dias J.A., Rodrigues J.J., Shu L., Ullah S. (2014). Performance evaluation of a cooperative reputation system for vehicular delay-tolerant networks. Eurasip J. Wirel. Commun. Netw..

[39] Wahab O.A., Otrok H., Mourad A. (2014). A dempster–shafer based tit-for-tat strategy to regulate the cooperation in vanet using qos-olsr protocol. Wirel. Pers. Commun..

[40] Al-Terri D., Otrok H., Barada H., Al-Qutayri M., Al Hammadi Y. (2017). Cooperative based tit-for-tat strategies to retaliate against greedy behavior in VANETs. Comput. Commun..

[41] Charilas D.E., Georgilakis K.D., Panagopoulos A.D. (2012). ICARUS: HybrId inCentive mechAnism for coopeRation stimUlation in ad hoc networkS. Ad Hoc Netw..

[42] Wang X., Cai Y., Li Z. (2014). A novel hybrid incentive mechanism for node cooperation in mobile cyber-physical systems. Int. J. Parallel Emergent Distrib. Syst..

[43] Gupta S.G., Ghonge M.M., Thakare P.D., Jawandhiya P.M. (2013). Open-source network simulation tools: An overview. Int. J. Adv. Res. Comput. Eng. Technol. (IJARCET).

